# Sarcospan: a small protein with large potential for Duchenne muscular dystrophy

**DOI:** 10.1186/2044-5040-3-1

**Published:** 2013-01-03

**Authors:** Jamie L Marshall, Rachelle H Crosbie-Watson

**Affiliations:** 1 Department of Integrative Biology and Physiology, University of California Los Angeles, 610 Charles E. Young Drive East, Terasaki Life Sciences Building, Los Angeles, CA, 90095, USA; 2 Center for Duchenne Muscular Dystrophy, University of California Los Angeles, Los Angeles, CA, 90095, USA; 3 Molecular Biology Institute, University of California Los Angeles, Los Angeles, CA, 90095, USA

**Keywords:** Akt, Cell adhesion, Duchenne, Dystrophin, Integrin, Laminin-binding, *mdx*, Muscular dystrophy, Neuromuscular junction, Regeneration, Sarcolemma, Sarcospan, Utrophin

## Abstract

Purification of the proteins associated with dystrophin, the gene product responsible for Duchenne muscular dystrophy, led to the discovery of the dystrophin-glycoprotein complex. Sarcospan, a 25-kDa transmembrane protein, was the last component to be identified and its function in skeletal muscle has been elusive. This review will focus on progress over the last decade revealing that sarcospan is an important regulator of muscle cell adhesion, strength, and regeneration. Investigations using several transgenic mouse models demonstrate that overexpression of sarcospan in the mouse model for Duchenne muscular dystrophy ameliorates pathology and restores muscle cell binding to laminin. Sarcospan improves cell surface expression of the dystrophin- and utrophin-glycoprotein complexes as well as α7β1 integrin, which are the three major laminin-binding complexes in muscle. Utrophin and α7β1 integrin compensate for the loss of dystrophin and the finding that sarcospan increases their abundance at the extra-synaptic sarcolemma supports the use of sarcospan as a therapeutic target. Newly discovered phenotypes in sarcospan-deficient mice, including a reduction in specific force output and increased drop in force in the diaphragm muscle, result from decreased utrophin and dystrophin expression and further reveal sarcospan’s role in determining abundance of these complexes. Dystrophin protein levels and the specific force output of the diaphragm muscle are further reduced upon genetic removal of α7 integrin (Itga7) in SSPN-deficient mice, demonstrating that interactions between integrin and sarcospan are critical for maintenance of the dystrophin-glycoprotein complex and force production of the diaphragm muscle. Sarcospan is a major regulator of Akt signaling pathways and sarcospan-deficiency significantly impairs muscle regeneration, a process that is dependent on Akt activation. Intriguingly, sarcospan regulates glycosylation of a specific subpopulation of α-dystroglycan, the laminin-binding receptor associated with dystrophin and utrophin, localized to the neuromuscular junction. Understanding the basic mechanisms responsible for assembly and trafficking of the dystrophin- and utrophin-glycoprotein complexes to the cell surface is lacking and recent studies suggest that sarcospan plays a role in these essential processes.

## Review

### Identification of sarcospan

Muscular dystrophies represent a group of progressive muscle disorders characterized by extensive muscle wasting and weakness. Duchenne muscular dystrophy (DMD) is caused by mutations in the dystrophin gene that result in loss of dystrophin, a protein that is normally localized to the subsarcolemma [1-5]. Discovery of dystrophin-associated proteins, referred to as the dystrophin-glycoprotein complex (DGC), represent a major advancement in the understanding of the DGC’s function in skeletal muscle and provide further support for the contraction-induced sarcolemma injury model underlying DMD pathogenesis [1,2,4,5]. In addition to dystrophin, the DGC is composed of α/β-dystroglycan (DG), the sarcoglycans (SGs), the syntrophins, and dystrobrevin (for review, [6]). One of the last components of the DGC to be identified was a 25-kDa dystrophin-associated protein (DAP), which was resistant to identification, in part due to lack of polyclonal antibodies that cross-reacted with the 25-kDa DAP (also called A5) from goats and sheep, immunized with the DGC [7]. The hydrophobic probe, 3-trifluoromethyl-3-(*m*-^125^I] iodophenyl) diazirine or TID, bound very strongly to the 25-kDa DAP, suggesting that it might be an integral membrane protein [5]. In fact, TID binding to the 25-kDa DAP was greater than its binding to the SGs or β-DG, which possess a single transmembrane span, providing strong evidence that the 25-kDa DAP contained multiple membrane-spanning regions and was unlikely to be a protein degradation product, as had been speculated based on its weak staining with Coomassie-Brilliant blue [5,7]. Identification of the 25-kDa DAP was accomplished by in-gel digestion and sequencing of two amino acid peptides, leading to isolation of the corresponding human cDNA [7] that was previously identified in part as Kirsten ras associated gene (krag), a gene that is co-amplified with Ki-ras in the Y1 murine adrenal carcinoma cell line [8,9]. The gene was renamed to sarcospan (SSPN) for its multiple sarcolemma spanning helices predicted from hydropathy analysis [7]. While SSPN is expressed in many non-muscle tissues [10,11], its biochemical characterization was performed in skeletal muscle where it is most abundant.

### Rigorous criteria define components of the dystrophin-glycoprotein complex

Although characterization of the 25-kDa DAP led to its identification, there was much speculation that SSPN was a contaminant of the purified complex rather than a *bona fide* member of the DGC. Integral components of the DGC are defined by four biochemical characteristics and SSPN was rigorously tested with these established criteria. First, purification of the DGC from skeletal muscle membranes enriches proteins that are associated in a complex with dystrophin. Campbell and colleagues exploited the presence of several glycoproteins within the DGC to enrich the complex using succinylated wheat germ agglutinin (sWGA) lectin chromatography of digitonin-solubilized skeletal muscle membranes [1-5]. sWGA enrichments containing the DGC can be further purified by diethylaminoethyl (DEAE)-cellulose ion exchange chromatography, which separates the DGC from abundant calcium channels. It was discovered that SSPN elutes from DEAE columns at 175 mM NaCl, along with purified DGC components [7]. A second characteristic of DGC proteins is their migration as a complex during high-speed centrifugation through sucrose gradients. Only proteins that bind with high affinity and specificity will be retained with dystrophin during sucrose gradient fractionation where it migrates as an 18S complex [2-4,12]. SSPN co-migrates with peak DGC-containing fractions providing additional evidence that SSPN is an integral component of the DGC [5,7,13,14]. In contrast, while a fraction of caveolin-3 maintains association with the DGC during purification by sWGA lectin affinity chromatography, it is localized to heavier fractions during sucrose gradient centrifugation [7,13]. For the third criterion, the laminin binding capacity of α-dystroglycan (α-DG) was exploited to separate the DGC from other membrane-associated proteins. Application of sWGA enrichments from skeletal muscle reveals that SSPN is entirely retained on laminin-sepharose columns, but caveolin-3 is found only in the void fraction [13,15-19]. Finally, it is well established that core components of the DGC depend on dystrophin for localization to the sarcolemma. In dystrophin-deficient DMD patients and the *mdx* mouse model, SSPN is absent from the sarcolemma while membrane expression of caveolin-3 is not affected by loss of dystrophin [7,13].

### Structural analysis of sarcospan provides insight into function

**Figure 1:**
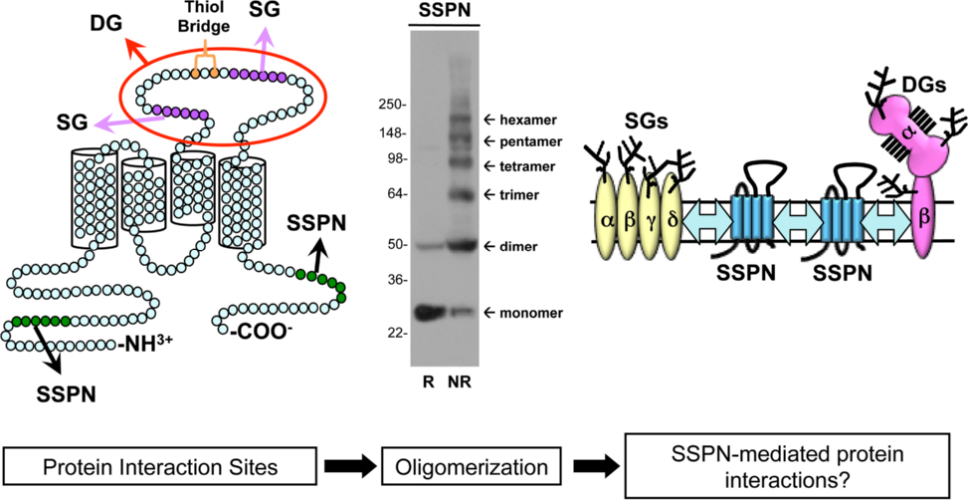
**SSPN interacts with the sarcoglycans and forms oligomers characteristic of tetraspanins.** SSPN is a tetraspanin-like protein, with four transmembrane domains, which complexes with the DGC and UGC at the sarcolemmal membrane of skeletal muscle. Site directed mutagenesis of SSPN revealed that the N- and C-termini as well as regions of the large extracellular loop (LEL, between transmembrane domains 3 and 4) are necessary for SSPN-SSPN and SSPN-SG interactions, respectively [24]. Deletion mutagenesis, in which regions of six amino acids were removed at a time, was performed to identify regions within SSPN that are important for protein interactions (left). The N-terminus and C-terminus (green) are critical for SSPN dimer formation and the LEL is important for trimer and tetramer oligomers. SSPN-SG interactions were identified in the LEL (purple) and mutations in the LEL disrupt SSPN monomer formation, likely due to disruption of thiol bonds (orange) critical for stabilizing the structure of SSPN. Immunoblot analysis of skeletal muscle lysates from SSPN transgenic mice demonstrates that SSPN forms homo-oligomers under non-reducing (NR) conditions (middle) [24]. In reducing conditions (R), SSPN exists solely in monomeric form. Similar to all tetraspanins, SSPN forms higher ordered structures through homo-oligomerization. We propose a model whereby SSPN-SSPN oligomers form a scaffold on which the DGC and UGC complexes are assembled (right). DGC, dystrophin-glycoprotein complex; DG, α/β dystroglycan; SG, sarcoglycans; UGC, utrophin-glycoprotein complex.

Topology algorithms predict that SSPN possesses four transmembrane domains with a small extracellular loop (between transmembrane domains 1 and 2), a large extracellular loop (LEL; between transmembrane domains 3 and 4), and intracellular N- and C-termini (Figure 1) [7]. Although several protein families contain four transmembrane domains, dendrogram analysis suggests that SSPN is related to the tetraspanins, although not all characteristics are conserved. Like other tetraspanins, the LEL of SSPN contains conserved Cys residues that are important for tertiary structure, although SSPN lacks the hallmark Cys Cys Gly motifs within the LEL and conserved sites for N-linked glycosylation and palmitolyation that are characteristic of tetraspanins [20]. Tetraspanins facilitate protein interactions by forming tetraspanin-enriched microdomains within the membrane to regulate intracellular cell signaling (for review, [20-23]). Similarly, SSPN forms higher ordered homo-oligomers by laterally associating with one another in the sarcolemma of skeletal muscle (Figure 1) [24].

Reconstitution of SSPN oligomerization using a heterologous cell expression system and muscle lysates from SSPN transgenic mice reveals the presence of pentamers that were maintained during high-speed ultracentrifugation through non-reducing sucrose gradients (Figure 1) [24]. Using a site-directed mutagenesis approach, SSPN-SSPN interfaces were defined within the intracellular (N- and C-termini) and extracellular regions of SSPN, suggesting that the formation of SSPN oligomers occurs through a complex set of protein interactions (Figure 1). Alanine replacement of cysteine residues reveals that intramolecular thiol bridges between Cys 162 and Cys 164 within the LEL are critical determinants of SSPN structure [24]. In fact, mutation of any cysteine within the LEL disrupts cysteine packing within the LEL, leading to destabilization of SSPN monomer formation [24]. Based on structural and functional analysis of SSPN overexpression in several mouse models, it is reasonable that multiple SSPN proteins may interact with each adhesion complex, thereby mediating cross-talk between transmembrane glycoprotein complexes. The biological significance of SSPN oligomers mediating protein-protein interactions between adhesion complexes is appealing, but requires further investigation.

Although SSPN exhibits many tetraspanin-like characteristics, it may be more structurally similar to the CD20 family of proteins, which includes the beta subunit of the high affinity receptor for IgE Fc [25]. Members of the CD20 family span the plasma membrane four times and possess a LEL (between transmembrane domains 3 and 4) as well as intracellular N- and C-termini [26-29]. The regions of homology that define the CD20 family are largely within the transmembrane domains. Similar to tetraspanins, CD20 forms multimeric oligomers within the plasma membrane and is unlikely to exist solely in a monomeric state [30]. The specific function of CD20 has not been elucidated, but CD20 localizes to lipid rafts where it may play a role in regulating cell cycle progression, tyrosine kinase-dependent signaling, and B-cell differentiation (for review, [31]).

### Sarcospan and the sarcoglycans form a subcomplex

The SGs are single pass transmembrane glycoproteins referred to as α-, β-, γ-, and δ-SG (for review, [6]). The first evidence that the DGC is composed of biochemically distinct subcomplexes came from experiments in which purified DGC was subjected to alkaline conditions that dissociate pH-sensitive protein interactions [4]. The finding that SSPN tightly associates with the SG subcomplex is supported by sucrose gradient analysis of alkaline-treated preparations revealing that SSPN co-sediments exclusively with the SGs [32]. Furthermore, treatments with denaturing agents such as sodium dodecyl sulfate [33] and n-octyl β-D-glucoside [34] fail to disrupt the integrity of the SG-SSPN subcomplex. Finally, the SG-SSPN subcomplex can be reconstituted in an *in vivo* cell culture model system lacking expression of DGs and dystrophin [32]. SSPN also associates with the SGs in smooth muscle purified from kidney and lung tissues [35-38] as well as epididymal white adipose tissue [11].

Mutations in the α-, β-, γ-, or δ-SG genes cause autosomal recessive limb-girdle muscular dystrophy (AR-LGMD) type 2D, 2E, 2C, and 2F, respectively (for review, [39]) leading to absence or a significant reduction in the SG subcomplex from the sarcolemma (for review, [40]). Genetic ablation of individual SG genes in mice has generated robust animal models for the SG-deficient AR-LGMDs in which the entire SG complex is absent from the sarcolemma (for review, [40]). Similarly, a large deletion in the δ-SG gene causes cardiomyopathic and myopathic features in the BIO14.6 hamster model [41,42]. Consistent with its tight biochemical association with the SGs, SSPN is absent from the sarcolemma of mice deficient in α-, β-, and δ-SG as well as the δ-SG deficient BIO14.6 hamster [32,36,43-46]. SSPN expression is restored to normal levels in BIO14.6 muscle after delivery of an adenovirus encoding δ-SG [32]. Furthermore, investigation of over 30 AR-LGMD muscle biopsies with primary mutations in α-, β-, or γ-SG genes that result in either complete or partial absence of the SGs revealed that SSPN was absent from the sarcolemma [46]. The levels of SSPN expression were not analyzed in γ-SG deficient mice [47], but it would be interesting to determine if the trend was similar to the observations made in human AR-LGMD biopsies. Interaction between the SGs and SSPN is very sensitive to structural perturbations within the LEL of SSPN, as revealed by alanine scanning and deletion mutagenesis within the LEL (Figure 1) [24]. SSPN interaction with the SGs is not unique to skeletal muscle. The SSPN-SG subcomplex has been characterized in many tissues, including the smooth muscle from lung and kidney [11,35,48-51]. In this context, SSPN interacts with a modified SG subcomplex consisting of β-, γ-, and ε-SG (a homolog of α-SG). Interestingly, the SSPN-SG subcomplex does not co-migrate in sucrose gradient fractions of sWGA purified DG from epithelial cells derived from lung or kidney tissue [35]. SSPN is not conserved in *Drosophila melanogaster* and *Caenorhabditis elegans*, thus the resulting DGC equivalent lacks SSPN and is predicted to be composed of DGs, SGs, and dystrophin [52,53].

### Sarcospan uniquely increases abundance of laminin-binding complexes

**Figure 2:**
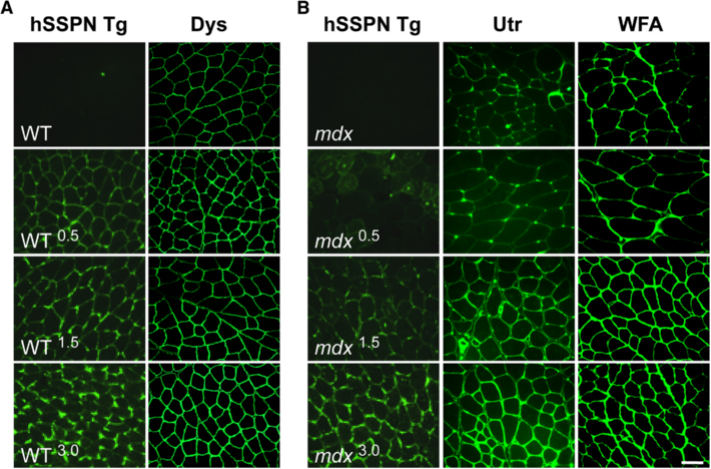
**SSPN increases abundance of laminin-binding complexes at the sarcolemma.** Several lines of SSPN-transgenic mice with 0.5-, 1.5-, and 3-fold levels of SSPN overexpression were generated to investigate the dose-dependent effects of SSPN expression. SSPN transgenic mice on C57/Bl6 background (WT, WT^0.5^*,* WT^1.5^, WT^3.0^) as well as 0.5-, 1.5-, and 3-fold SSPN transgenic on *mdx* background (*mdx*, *mdx*^0.5^*, mdx*^1.5^, *mdx*^3.0^) were analyzed [19,70]. (**A**) Transverse cryosections of quadriceps muscle from six-week old SSPN-transgenic mice were stained with antibodies to dystrophin (Dys) and hSSPN to reveal exogenous SSPN expression (hSSPN Tg). (**B**) Muscle sections from SSPN-transgenic *mdx* mice were also stained with utrophin (Utr) and hSSPN. Sections overlayed with *Wisteria floribunda* agglutinin (WFA) lectin reveal increased cell surface glycosylation with elevations in SSPN overexpression [19]. WFA lectin binds terminal GalNAc residues and serves as a marker for the CT antigen modification of α-DG that normally occurs at the NMJ [19]. Note that SSPN increases cell surface expression of dystrophin, utrophin, and glycosylation in a manner dependent on SSPN abundance. Bar, 50 μm. CT, cytotoxic T cell; NMJ, neuromuscular junction.

**Figure 3:**
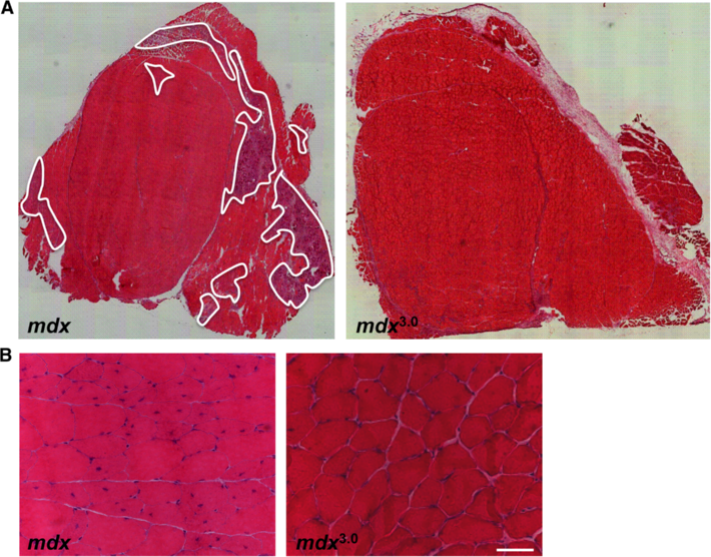
**SSPN overexpression ameliorates dystrophic pathology in *mdx *mice.** Transverse cryosections of quadriceps muscle from six-week old *mdx* and SSPN transgenic *mdx* (*mdx*^3.0^) mice were stained with hematoxylin and eosin (H&E) and visualized at low magnification to reveal the extensive areas of necrosis, denoted in white, in *mdx* muscle (**A**). Necrosis was significantly diminished in SSPN transgenic *mdx* muscle [69]. Sections were also viewed at higher magnification for evaluation of degeneration/regeneration, which is marked by central nucleation of myofibers (**B**). Overexpression of SSPN in *mdx* muscle dramatically reduced central nucleation [69]. Bar, 50 μm.

The functional replacement of utrophin for dystrophin is one of many attractive therapeutic strategies for the treatment of Duchenne muscular dystrophy. Utrophin is an autosomal homolog of dystrophin and forms a functionally similar utrophin-glycoprotein complex (UGC) where utrophin replaces dystrophin [54-56]. In normal muscle, the UGC is located at the postsynaptic membrane of neuromuscular junctions (NMJ) [55,57,58]. Overexpression of both full-length utrophin and mini-constructs ameliorates dystrophic pathology in the *mdx* mouse model of DMD [59-68]. Mice engineered to overexpress threefold levels of human SSPN driven by the muscle-specific human skeletal actin promoter increased ectopic expression of utrophin, dystrophin, and α7β1 integrin at the sarcolemma (Figure 2) [19,69]. In fact, analysis of SSPN transgenic mice overexpressing 0.5- and 1.5-fold levels of SSPN reveals that these molecular events occur in a SSPN dose-dependent manner (Figure 2). Introduction of SSPN (threefold) ameliorates dystrophy in the *mdx* mouse model of DMD by reducing cycles of degeneration/regeneration and preventing sarcolemma damage (Figure 3). Biochemical purification of the UGC complex using lectin affinity chromatography followed by sucrose gradient ultracentrifugation revealed that SSPN is a component of the UGC [19,69]. Consistent with the role of SSPN in regulating adhesion complexes at the cell surface, overexpression of 10-fold levels of SSPN causes formation of insoluble protein aggregates at the sarcolemma, resulting in muscle pathology [70]. To date, thirteen human SSPN transgenic lines have been created and only one line expressed tenfold levels of SSPN. In both 3- and 1.5-fold lines of SSPN expression, internal down regulation of endogenous SSPN was observed, suggesting that the levels of SSPN are tightly controlled within the cell [19]. Based on this data, it may be unlikely to achieve tenfold levels of SSPN in a clinical setting. Future studies are needed to determine whether SSPN amelioration of dystrophic pathology occurs in aged *mdx* mice and whether SSPN ameliorates disease in mouse models of laminin-deficient congenital muscular dystrophy and SG-deficient LGMD.

### Sarcospan affects glycosylation of α-dystroglycan

The cytotoxic T cell (CT) GalNAc transferase (Galgt2) is confined to the NMJ and catalyzes addition of the terminal β1,4 GalNAc residues onto the CT carbohydrate of a subset of α-DG proteins [71,72]. α-DG is the predominant glycoprotein modified with the CT carbohydrate in skeletal muscle where it is enriched at the postsynaptic membrane of the NMJ [73]. Overexpression of Galgt2 in *mdx* mice increases abundance and extrasynaptic expression of α-DG modified with the CT antigen, resulting in improved laminin-binding activity [72,73]. Overexpression of SSPN in *mdx* mice increases GalNAc modifications in a similar manner to the overexpression of Galgt2, as revealed by the increased cell surface binding of the lectin *Wisteria floribunda* agglutinin (WFA) [19], which is a marker for NMJ-specific CT carbohydrate modification of α-DG (Figure 2) [72,74-77]. WFA binding is localized to NMJs in normal muscle and is increased around the extra-synaptic sarcolemma of *mdx* muscle cryosections [19,78]. WFA binding to SSPN-transgenic *mdx* (*mdx*^3.0^) muscle was significantly increased around the extra-synaptic sarcolemma similar to utrophin expression [19]. Increased Galgt2 activity in *mdx* mice results from a two-fold elevation of *Galgt2* mRNA levels in *mdx* muscle relative to wild-type controls [73]. However, SSPN does not affect *Galgt2* transcript abundance, raising the possibility that SSPN increases Galgt2 activity or improves α-DG as a substrate for Galgt2 [19]. SSPN also increases laminin at the sarcolemma as well as levels of plectin-1, which binds cytoskeletal proteins including β-DG, dystrophin, utrophin, and F-actin [79-82], supporting the conclusion that SSPN strengthens the structural connection between actin and laminin across the sarcolemma [19]. Furthermore, laminin binding to α-DG was restored to normal levels in threefold SSPN overexpressing *mdx* muscle [19]. Transgenic overexpression of 0.5- and 1.5-fold levels of SSPN increased glycosylation of α-DG, Akt signaling, and utrophin levels, but failed to restore laminin binding or reduce muscle degeneration/regeneration, revealing a minimum (threefold) level of SSPN needed for ‘rescue’ (Figure 2) [19].

The ‘dystroglycanopathies’ are a group of disorders resulting from hypoglycosylation of α-DG that abolishes its laminin-binding function. A spontaneous mutation in the *LARGE* gene, which encodes an enzyme with xylosyltransferase and glucuronyltransferase activities, causes muscular dystrophy in the myodystrophy (*myd*) mouse [83]. In *myd* muscle, α-DG is hypoglycosylated and exhibits severely reduced ligand binding activity due to loss of the glycan-laminin binding domain on α-DG [84,85]. LARGE elongates phosphorylation dependent glycosylaminoglycan modifications on the central mucin domain of α-DG by direct interaction with α-DG [86,87]. Loss of LARGE increases utrophin and SSPN staining and WFA binding around the extra-synaptic sarcolemma of *myd* muscle [19]. Introduction of the SSPN transgene into skeletal muscle of *myd* mice further elevated WFA binding along with broad, extra-synaptic localization of utrophin, while removal of SSPN from *myd* muscle reduced utrophin and GalNAc-glycan modification of α-DG [19]. Pathology of *myd* muscle was unaffected by the loss of SSPN or SSPN overexpression, demonstrating that alterations in GalNAc glycosylation of α-DG or utrophin abundance do not affect absence of the main laminin-binding domain on α-DG [19]. The conclusion from these experiments is that Galgt2 (or enzyme with similar function) modifies α-DG in the absence of ‘LARGE’ glycans, demonstrating that GalNAc modification of α-DG can occur independently of O-mannose-linked glycans. Additionally, these data reveal that GalNAc carbohydrate structures on α-DG are unable to compensate for the loss of LARGE glycans, which constitute the major laminin-binding motif.

### A newly discovered phenotype for sarcospan-null mice

Evidence that SSPN loss may affect skeletal muscle was first suggested from comparative microarray analysis revealing a mild decrease in mRNA levels of dystrophin and α-SG in SSPN-null muscle [88]. Furthermore, the authors reported increased expression of two genes, osteopontin and the S100 calcium-binding protein calgranulin B, that have been implicated in immunological function and fibrosis [89-91]. A second gene expression study compared hippocampus and cortex of mice exposed to chronic constant hypoxia (CCH) and chronic intermittent hypoxia (CIH). CCH occurs in chronic lung diseases or at high altitudes while CIH develops from disorders such as sleep apnea or sickle cell disease. SSPN was one of two identified genes down-regulated in the hippocampus and cortex after both treatments [92]. SSPN knockdown in a cultured glioma cell line (LN-229) did not affect cell division as determined by bromodeoxyuridine (BrdU) incorporation, but did increase vulnerability of glioma cells to hypoxia [92].

Genetic ablation of SSPN did not appear to alter muscle physiology or strength in young mice [93]. However, when SSPN-null mice were analyzed at older ages (4.5 month old), several deficiencies emerged. Reduction in the levels of the UGC and DGC as well as diminished NMJ-specific glycosylation of α-DG and decreased laminin-binding was observed in aged SSPN-nulls [14]. Diaphragms from older SSPN-null mice exhibited diminished specific force generating capacity and were more susceptible to eccentric-contraction induced damage as evidenced by the increased percentage drop in force compared to wild-type controls [14]. These physiological phenotypes were not observed in extensor digitorum longus (EDL) or soleus muscles, suggesting that decreased DGC expression in EDL or soleus muscles is insufficient to manifest in loss of muscle strength or sarcolemma damage [14,93].

### Sarcospan genetically interacts with integrins

It is well established that all tetraspanins interact with integrin partners to regulate cell signaling, adhesion, and laminin-binding capacity of integrins (for review, [94,95]). The primary integrin expressed in adult skeletal muscle is α7β1 integrin, whichis localized at the NMJ and myotendinous (MTJ) regions within the sarcolemma [96-99]. The relatively mild phenotype of *mdx* mice has been attributed to increased, compensatory expression of both utrophin and α7β1 integrin in response to loss of dystrophin that, when ablated, exacerbates *mdx* pathology [100-102]. Overexpression of Itga7 in *mdx*:utrophin-null mice and β1 integrin in *mdx* mice ameliorates pathology [103-105]. The recent observation that α7β1 integrin levels are increased in response to SSPN deficiency is intriguing as it suggests that α7β1 integrin compensates for SSPN function and moderates the severity of muscle phenotypes in SSPN-nulls [14]. Analysis of SSPN-deficient and Itga7-deficient double knockout (DKO) mice supports this hypothesis. In comparison to controls at 4.5-months of age, DKO mice exhibit increased kyphosis and premature lethality at one month of age, which is significant given that the single-nulls display no overt signs of pathology or lethality (Table 1) [14]. Furthermore, DKO muscle appears severely dystrophic with extensive fibrosis surrounding individual hypertrophic muscle fibers, in a manner identical to histological images of DMD biopsies [14]. Genetic removal of Itga7 from SSPN-nulls further reduced levels of the DGC at the sarcolemma, diminished laminin-binding to α-DG, and consequently decreased specific force output in the diaphragm (Table 1) [14]. The conclusion from this work is that SSPN is a necessary component of dystrophin and utrophin function and that SSPN modulation of integrin signaling is required for growth, extracellular matrix attachment, and muscle force development.

**Table 1:** Sarcospan- and α7 integrin-double nulls display severe growth and muscle phenotypes

**Genotype**	**Survival Analysis**^**a**^	**Kyphosis**^**a**^	**Myofiber CSA**^**a**^	**Central Nucleation**^**a**^	**Utrophin**^**a**^	**Dystrophin**^**a**^	**Integrin**^**a**^	**Laminin-Binding**^**a**^	**Signaling**^**a**^	**Specific Force**^**a**^
Wild type	100%	No	Normal	Normal	100%	100%	100%	100%	P-Akt/Akt: 100%	Normal (EDL, Soleus, Diaphragm)
									P-IGFR/IGFR: 100%	
SSPN null	100%	No	Normal	Normal	31%	47%	293%	69%	P-Akt/Akt: 49%	Normal (EDL, Soleus); Decrease (Diaphragm)
									P-IGFR/IGFR: 80%	
Itga7 null	95%	Minor	Normal	Normal	102%	48%	Absent	85%	P-Akt/Akt: 95%	Decrease (Diaphragm)
									P-IGFR/IGFR: 99%	
DKO	60%	Severe	Decrease	Increase	44%	34%	Absent	28%	P-Akt/Akt: 40%	Severe Decrease (Diaphragm)
									P-IGFR/IGFR: 35%	

^a^Measurements represented relative to wild type. DKO, double knockout; Itga7, α7 integrin.

The table summarizes phenotypic and biochemical data from 4.5-month old SSPN-null, Itga7-null, and SSPN-null:Itga7-null double knockout (DKO) mice. All comparisons are relative to age-matched wild-type mice. Survival studies were carried out to eight months of age. At this time point, 40% of the DKO mice had succumbed to death compared to less than 5% of the controls. The extent of kyphosis was documented in 4.5-month and 6-month old mice. Itga7-null mice exhibited minor kyphosis and the additional loss of SSPN caused severe kyphosis in DKO mice. Myofiber cross-sectional area (CSA) quantified from the quadriceps and diaphragm muscles at 4.5 months of age are represented. DKO muscles exhibited an increase in myopathy and increase in very small (0 to 500 μm^2^) myofibers. Central nucleation is provided as an indicator of overall muscle phenotype. Analysis of sarcolemmal damage (Evans blue dye assay) and fibrosis (Van Geison) of the quadriceps and diaphragm muscles at 4.5 months of age are exacerbated in DKOs [14]. The levels of utrophin, dystrophin, laminin-binding to α-DG, and β1 integrin were analyzed by densitometry of sWGA eluates from digitonin-solubilized total skeletal muscle lysates. SSPN-deficient mice exhibit a reduction in the UGC, DGC, and laminin-binding to α-DG and a corresponding compensatory increase in β1 integrin [14]. Loss of Itga7 results in a reduction in the levels of the DGC and laminin-binding to α-DG. Importantly, combined loss of Itga7 and SSPN causes further reduction of the DGC, UGC, and laminin-binding to α-DG compared to all controls. The relative levels of P-Akt/Akt and P-IGFR/IGFR are provided. Specific force measurements of the diaphragm muscles reveal a loss of specific force in SSPN-null and Itga7-null that is additive in DKO mice. Interestingly, specific force production of the EDL and soleus muscles are unaffected by the loss of SSPN suggesting that, in these muscles, integrin may successfully compensate for DGC or that reduction of the DGC does not affect EDL and soleus muscle physiology.

### Sarcospan activates Akt signaling pathways to facilitate regeneration

**Figure 4:**
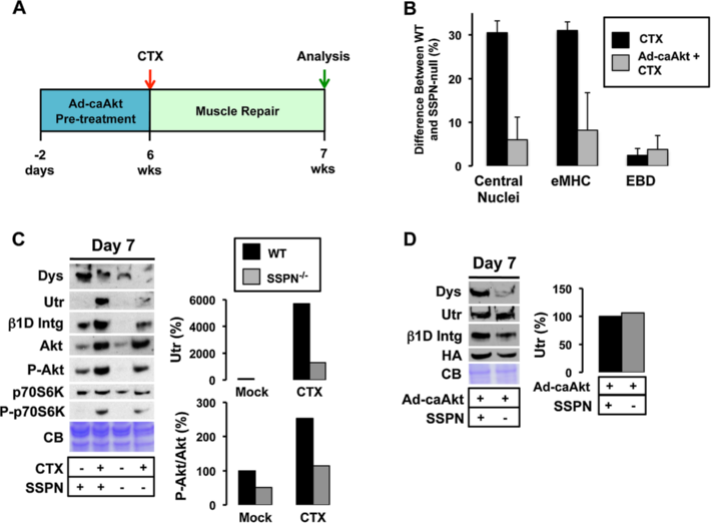
**Muscle recovery from cardiotoxin injury requires SSPN-dependent Akt activation.** (**A**) An acute injury model was used to investigate muscle repair in wild-type and SSPN-null mice. Quadriceps injected with equivalent volumes of saline (mock) and cardiotoxin dissolved in saline (CTX) were evaluated. CTX causes localized regions of myofiber necrosis followed by regeneration (for review, [125]). Mice at six weeks of age were injected with CTX (or mock) and analyzed after seven days. To test the dependency of muscle repair on Akt signaling, a subset of mice was pre-treated with adenovirus containing constitutively active Akt (Ad-caAkt) two days prior to CTX treatment. (**B**) Analysis was performed using H&E, laminin/eMHC, and laminin/EBD stained sections from quadriceps muscle. SSPN-deficient mice exhibit increased active regeneration (eMHC, 30%) seven days after CTX injury when wild-type mice have already undergone successful repair (black). Administration of Ad-caAkt prior to CTX treatment rescued the repair defect in SSPN-deficient mice (grey). (**C**) Immunoblot analysis of RIPA quadriceps lysates revealed reductions in the levels of dystrophin (Dys), utrophin (Utr), integrin (β1 integrin), phosphorylated Akt (P-Akt), and phosphorylated p70S6K (P-p70S6K) in SSPN-null mice compared to wild-type. Quantification of utrophin and P-Akt is provided. Coomassie blue (CB) serves as a loading control. (**D**) Immunoblot analysis of RIPA lysates from quadriceps pre-treated with Ad-caAkt revealed reductions in the levels of dystrophin (Dys) and integrin (β1 integrin) in SSPN-null mice compared to wild-type. Utrophin levels were restored in SSPN-null mice compared to wild-type mice with supplementation of active Akt (right). These data reveal that SSPN is upstream of the Akt signaling pathway regulating utrophin expression. The Ad-caAkt contains the HA-tag for detection [19]. RIPA, radioimmunoprecipitation assay.

**Figure 5:**
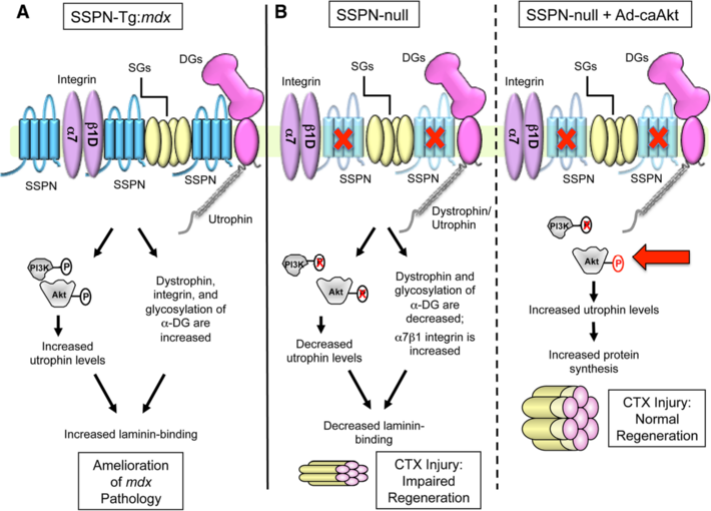
**Sarcospan is a critical regulator of laminin-binding receptors in muscle.** (**A**) The DGC/UGC and α7β1 integrin at the sarcolemma in *mdx*^3.0^ (SSPN-Tg:*mdx*) muscle is depicted. The dystroglycans (DGs; pink), sarcoglycans (SGs; yellow), sarcospan (SSPN; blue), dystrophin (grey) and integrins (purple) are shown. Overexpression of SSPN in *mdx* muscle elicits a series of molecular events that lead to restoration of laminin binding, amelioration of pathology, and restoration of membrane integrity [19]. As shown in the illustration, SSPN activates Akt, which stabilizes utrophin, and increases the abundance of integrin and WFA-reactive α-DG at the cell surface. SSPN facilitates increased CT antigen modification of α-DG and enhances transportation of utrophin-DG at the sarcolemma [19]. Collectively, these events lead to stabilization of the sarcolemmal membrane and amelioration of dystrophic pathology. (**B**) SSPN-null muscle exhibits decreased dystrophin and Akt activation followed by decreased expression of utrophin, resulting in the reduction of laminin-binding to α-DG (middle panel) [19]. Acute muscle injury by cardiotoxin injection into SSPN-null muscle impairs muscle regeneration (right) [19]. However, pre-treatment of SSPN-null mice with adenovirus containing constitutively active Akt (Ad-caAkt) restored the activation of downstream p70S6K, utrophin expression, and improved muscle regeneration (right panel). These studies reveal the importance of sarcospan and Akt in regulating utrophin expression that is critical for muscle repair. CT, cytotoxic T cell; DGC, dystrophin-glycoprotein complex; UGC, utrophin-glycoprotein complex; WFA, *Wisteria floribunda* agglutinin.

Modulation of the phosphatidylinositol-3 kinase (PI3K)/Akt signaling pathway leading to downstream activation of p70S6K protein synthesis pathways is important for regulation of muscle strength, hypertrophy, and pathophysiology (for review, [106,107]). The Akt signaling cascade through p70S6K is activated by several receptors, including: insulin like growth factor 1 (IGF-1) and β1 integrin associated integrin-linked kinase (ILK) (for review, [106,107]). Overexpression of constitutively active Akt in skeletal muscles of dystrophin-deficient *mdx* mice results in increased abundance of utrophin and α7β1 integrin, which leads to improvements in force generation [108,109]. Interestingly, overexpression of threefold levels of SSPN results in amelioration of *mdx* dystrophic pathology through stabilization of the UGC and α7β1 integrin at the sarcolemma, and activation of Akt and downstream p70S6K [19,70]. Conversely, the Akt/p70S6K pathway and activation of the IGF receptor is depressed in SSPN-deficient mice, rendering the muscle unable to repair efficiently after cardiotoxin-induced injury (Figure 4) [19]. Pretreatment of SSPN-null muscle with adenovirus expressing constitutively active Akt increased the UGC to normal levels and restored muscle regeneration after cardiotoxin-injury (Figure 4) [19]. The conclusion from these experiments is that SSPN modulates utrophin protein levels at least in part through Akt/p70S6K signaling pathways (Figure 5).

### A chaperone-like function for sarcospan is emerging

It has been assumed that the prematurely truncated dystrophin protein produced from the *mdx* mutation is rapidly degraded in muscle based on lack of its detection in whole skeletal muscle extracts. However, recent work has revealed that truncated dystrophin protein is synthesized in *mdx* mice [19]. In fact, truncated dystrophin proteins are produced and detected in high abundance in ER/golgi compartments in *mdx* muscle, suggesting that they accumulate in intracellular compartments due to insufficient transportation to the cell surface [19]. These data are exciting as they reveal for the first time that truncated dystrophin fragments are synthesized in *mdx* muscle, but then retained in intracellular membrane compartments rather than properly transported to the sarcolemma.

In addition to its role at the cell surface, a role for SSPN within the ER/golgi is suggested from recent studies. Biochemical analysis of ER/golgi membranes isolated from *mdx* muscle revealed abundant levels of utrophin and α-DG relative to wild-type [19]. Interestingly, utrophin and α-DG are reduced in ER/golgi preparations from SSPN transgenic *mdx* muscle while these same proteins are increased in abundance at the sarcolemma, suggesting that SSPN possesses chaperone-like functions to improve protein folding and/or trafficking to the cell surface (Figure 5).

### Sarcospan as a candidate disease gene

The sarcospan gene is localized to human chromosome 12p11.2 and is encoded by three small exons that are separated by very large introns [8,9]. A novel exon 4 was recently identified to encode for an alternative C-terminal region in humans. In fact, alternate mRNA splicing of human SSPN exons 1 and 2 to exon 4 generates a protein called microspan (μSPN) that lacks transmembrane domains 3 and 4 as well as the LEL so that the resultant protein has only two transmembrane spans and a novel intracellular C-terminus [10]. μSPN does not interact with the DGC and its expression is maintained in dystrophin-deficient muscle. Although μSPN is not localized to the sarcolemma, it is enriched in the sarcoplasmic reticulum (SR) [10]. Overexpression of μSPN in skeletal muscle of transgenic mice reduces levels of ryanodine receptor, dihydropyridine receptor as well as SERCA-1 resulting in aberrant triad morphology [10]. μSPN is also reduced in isolated SR membranes of δ-SG-null muscle contributing to SERCA dysfunction [110]. Given that both SSPN and μSPN interact with proteins that are critical to skeletal muscle function, it can be hypothesized that genetic mutations affecting SSPN function would have significant consequences for muscle. PCR-based approaches to screen muscular dystrophy patients for possible abnormalities within the SSPN gene have not yet produced any disease-causing mutations, although several single-nucleotide polymorphisms were identified in exons 2 and 3 [111]. SSPN was also excluded as a candidate disease gene for congenital fibrosis of the extraocular muscle (CFEOM), which is an autosomal dominant disorder linked to the pericentromere of chromosome 12 [112,113]. These findings may come as no surprise given the apparently normal phenotype of SSPN-deficient mice [93]. However, re-analysis of SSPN-deficient mice revealed significant phenotypes in muscle of aged mice and after exposure to conditions of cellular stress that may have implications for disease (Figure 5) [14,19]. Furthermore, the idea that SSPN may serve as a chaperone to improve cell surface expression of the DGC and UGC make it an excellent candidate as a genetic modifier of disease.

## Conclusions

Overexpression of many proteins and compounds ameliorates dystrophic pathology in the *mdx* mouse by increasing UGC abundance at the extrasynaptic sarcolemma. A sampling of these includes: CT GalNAc transferase (*Galgt2*) [73], ADAM12 [114], heregulin [115], L-arginine [116,117], activated calcineurin-A alpha [118], N-acetylcysteine [119-121], activated Akt [108,109], GW501516 (activates PPAR beta/delta) [122], artificial gene Jazz [123], and biglycan [124]. Several studies have now revealed that SSPN can be added to this list of secondary proteins that modify utrophin expression [19,69]. The mechanism by which SSPN increases expression of utrophin involves activation of Akt signaling and increased glycosylation of α-DG, likely by increased modification of *Galgt2*[19]. Introduction of constitutively active Akt or *Galgt2* alone also improves extrasynaptic utrophin expression, strongly suggesting that SSPN, Akt, and *Galgt2* may act *via* a common or overlapping pathway(s). It will be important to determine whether every gene that increases utrophin expression also alters Akt and α-DG glycosylation, which would provide further evidence for a common post-transcriptional mechanism controlling utrophin abundance. Furthermore, these data reveal that there are multiple targets that affect utrophin, which is encouraging for pharmacological and gene-based therapies. SSPN also increases expression of Itga7, and future studies will determine whether Itga7 and/or utrophin are required for SSPN’s ‘rescue’ effect. It is also critical to investigate whether Itga7 levels are affected by the many other genes that increase utrophin expression, which would reveal important mechanisms regulating laminin-binding receptors in skeletal muscle. SSPN is a promising therapeutic target, particularly for adeno-associated virus delivery due to its small size and low potential for unwanted immune reaction. Future studies will reveal the potential of this small protein to alleviate the significant problem of DMD.

## Abbreviations

BTX: bungarotoxin; CCH: chronic constant hypoxia; CIH: chronic intermittent hypoxia; CSA: cross-sectional area; CT: cytotoxic T cell; DAP: dystrophin-associated protein; DEAE: diethylaminoethyl; DG: dystroglycan; DGC: dystrophin-glycoprotein complex; DKO: double knockout; DMD: Duchenne muscular dystrophy; Dys: dystrophin; EBD: Evans blue dye; EDL: extensor digitorum longus; IGF-1: insulin-like growth factor; ILK: integrin-linked kinase; Intg: integrin; LEL: large extracellular loop; NMJ: neuromuscular junction; PCR: polymerase chain reaction; SDS: sodium dodecyl sulfate; SG: sarcoglycan; SSPN: sarcospan; UGC: utrophin-glycoprotein complex; Utr: utrophin; WFA: *Wisteria floribunda* agglutinin; sWGA: succinylated wheat germ agglutinin; μSPN: microspan.

## Competing interests

The authors declare that they have no competing interests.

## Authors’ contributions

Both authors contributed to writing and preparation of figures for the manuscript. All authors have read and approved the final manuscript.

## Authors’ information

Rachelle H. Crosbie-Watson, Ph.D. is a Professor of Integrative Biology and Physiology at the University of California Los Angeles and her research group is focused on investigation of macromolecular adhesion complexes at the cell surface in normal and dystrophic skeletal muscle. Professor Crosbie-Watson earned a Ph.D. in biochemistry from the University of California Los Angeles investigating structure-function relationships of contractile proteins and she identified sarcospan during her MDA-sponsored postdoctoral fellowship at the University of Iowa College of Medicine with Professor Kevin P. Campbell (HHMI).

Jamie L. Marshall, Ph.D. is a postdoctoral researcher in Dr. Crosbie-Watson’s laboratory at the University of California Los Angeles. Dr. Marshall earned a Ph.D. in Molecular, Cellular, and Integrative Physiology from the University of California Los Angeles investigating the role of sarcospan in the adhesion glycoprotein complexes at the sarcolemma. Dr. Marshall pioneered purification the utrophin-glycoprotein complex [69] and discovered novel deficits of this complex in sarcospan-deficient muscle [14,19,]. Dr. Marshall has received numerous predoctoral fellowships as well as a postdoctoral fellowship based on these studies. Dr. Marshall has extensive experience in genetics, analysis of dystrophic mouse models, and detailed biochemical investigation of the dystrophin- and utrophin-glycoprotein complexes.
